# Training Nursing Skills: A Quantitative Study of Nursing Students' Experiences before and after Clinical Practice

**DOI:** 10.1155/2018/8984028

**Published:** 2018-03-11

**Authors:** Elisabeth Solvik, Solveig Struksnes

**Affiliations:** Faculty of Medicine and Health Sciences, Department of Health Sciences Gjøvik, NTNU (Norwegian University of Science and Technology), Postboks 191, 2802 Gjøvik, Norway

## Abstract

**Introduction:**

Requirements for Patient Safety suggest that students encounter patients well prepared. In clinical laboratory practice (CLP), the students simulate patient situations as a preparation for internship. Various CLP models have been tried out to meet the students' prerequisites and learning strategies.

**Objectives:**

The purpose of this study was to try out two different learning sessions related to the bed bath procedure.

**Design and Methods:**

The study has a descriptive, quantitative design with elements from clinically controlled trials.

**Sample:**

The population of 160 first-year students was randomly divided into two classes.

**Questionnaires:**

Two questionnaires were answered with six-month intervals: Form 1 immediately after the first training session and Form 2 a short time after clinical practice.

**Findings:**

A majority of the nursing students reported that the exercises in the clinical lab were a good way to prepare for the practice, although most of them did not perceive that the procedure conducted at the university resembled how it is conducted in clinical practice. Age or level of discomfort related to organization of the skills training did not have impact on the students' confidence in mastering bed bath in clinical practice. Students without previous experience were less confident to master the procedure in clinical practice, but the results evened out during the internship.

**Conclusions:**

The results from this study could indicate that the students' age to a larger extent should be considered in the universities' facilitation of nursing students' clinical preparations, to improve the transition to “real life” as smoothly and meaningfully as possible to nursing students.

## 1. Introduction

The practice field is a significant learning arena for nursing students in Norway, as half of the bachelor's program takes place in clinical practice [1]. Thus, preparations for the students' meeting with real patients constitute a substantial part of teaching efforts within the university. The comprehension of the transfer value of these preparations when it comes to clinical practice probably has an impact on students' achievements in the field of practice [2]. It has been argued that nursing education is inadequate in preparing students for practice and contributes to burnout syndrome among nurses and an earlier retirement from the profession [3, 4].

Nursing students have various backgrounds and different prerequisites for goal achievement in accordance with the National Curriculum of Nursing [1]. Requirements for Patient Safety [5] suggest that students encounter patients well prepared and with the proper knowledge and practical skills required within an increasingly specialized healthcare. Consequently, several nursing education institutions have introduced clinical skills tests ahead of clinical practice periods.

At the Norwegian University of Science and Technology (NTNU), simulated patient scenarios are used to a large extent as preparation for the students' clinical studies in practice. In clinical laboratory practice (CLP) the students simulate patient situations at various levels, from basic simulation in which fellow students play the roles of “patient” and “nurse” to more advanced scenarios with technologically advanced simulators (manikins) [6, 7]. The practical exercises are usually organized with student groups (10–12) working together under the supervision of one lecturer per group. Each student experiences merely one supervised training per procedure, due to the fact that this is a resource intensive learning activity. A single training session is not sufficient to assure the level of the students' skills before passing the tests required to enter clinical practice. Hence, students are encouraged to familiarize themselves with the procedures before and after the organized CLP. The development of electronic textbooks, with evidence-based descriptions and instructive videos of relevant procedures, has been produced to support the students in these unsupervised study activities. It is uncertain to what extent this has been done. Various CLP models have been tried out, some of these in cooperation with nurses from the clinical fields [8, 9]. It is also questioned to what extent the preparations within the university should be extended, so that training sessions (simulation) can replace some of the time spent in clinical practice [10, 11].

The current project was completed for freshmen in bachelor's nursing in the spring of 2014 and was part of the CLP before the first clinical practice in nursing homes. This includes skills training in various procedures before their first practice period in community healthcare setting.

## 2. Objectives

The overall goal for CLP is to ensure that nursing students who enter their first clinical practice period in community healthcare settings have the proper defensible knowledge and practical skills required to take care of patients in a safe manner.

The purpose of this study was to try out two different learning sessions related to the bed bath procedure. Six research items were described:

1. To investigate the students' degree of satisfaction with the learning session's organization and relevance before and after practice
2. To compare the perceptions of the project group and the control group
3. To compare perceptions of students with different age, with and without previous clinical experience and with unequal extent of training
4. To explore whether there are correlations between the perceptions before and after the clinical practice period.

## 3. Design and Methods

The study has a descriptive, quantitative design, and contains elements that characterize clinically controlled trials [12]. It is a cross-sectional study in which data were collected using two questionnaires answered with six-month intervals.

The study was approved by the Norwegian Social Data Services (NSD). All nursing students in their first year of the bachelor's program were informed both orally and in writing about the project and the opportunity to participate, two–four weeks prior to implementation.

Participating in the training session was mandatory, but to deliver the questionnaires was voluntary.

### 3.1. Sample

The population of 160 students was randomly divided into two classes, Class 1 (*N* = 79) and Class 2 (*N* = 81). Class 1 was chosen to be a control group, while Class 2 was chosen to be the project group. Each class was divided into half so that the total was held in four training sessions.

### 3.2. Questionnaires

The forms were inspired by a questionnaire developed for the National League for Nursing [10]. This form was translated into Norwegian at NTNU in 2010, and it was in this connection that permission was given to use it further [13]. The instruments were translated from English into Norwegian and then translated back into English, according to Polit and Beck [12]. Both translators were bilingual.

Form 1 was completed immediately after the first training session, whereas Form 2 was filled out a short time after clinical practice (Table 1).

In both forms the respondents reported to what degree they agreed/disagreed with the statements, according to a five-point Likert scale. The value of one represents a high degree of disagreement with the statement and five represents a high degree of agreement.

Demographic data collected included the respondents' age, whether they had experience from practice before, and how many hours they had trained with the procedure in question before clinical practice.

The implementation was somewhat different for the two groups as regards preparation for the exercise and teaching role, which is outlined in Table 2.

The project group experienced a more problem-oriented and student-active approach than was the case in the control group. Thus, there were half as many supervisors attending, and the supervisors' roles were different within the two groups. In the control group the supervisors followed up the students' activity closely and presented verbal and practical guidance and solutions. The supervisors of the project group had a minimal role and were only available for questions related to helping to facilitate the training session by providing sufficient equipment. The least experienced lecturers were chosen for the sessions with the project groups, on the assumption that they could more easily take this role than the experienced supervisors. How the roles should be practiced was incidentally discussed with the supervisors for both groups, to ensure an approximately equal approach during the sessions.

### 3.3. Data Collection

Immediately after the exercise, the students in both groups were asked to fill out the questionnaire individually and put it at the designated place before they left the rehearsal hall. The second form (after clinical practice) was handed over to the supervisor in charge during the last week of clinical practice.

### 3.4. Data Analysis

The questionnaires were coded with numbers that represented the individual student and class affiliation, with the answers registered into SPSS, version 22. Frequency tables were set up, and both Mann–Whitney *U* test (two groups) and Kruskal–Wallis test (three groups) were conducted to compare statements between defined groups according to the research questions. Lastly, correlation tests (Pearson's *r*) were done to assess possible relationships between statements in Forms 1 and 2.

## 4. Results

Results are presented according to the research objectives.  Table 3 shows an overview of the demographic data of the respondents.

### 4.1. Students' Level of Satisfaction with the Clinical Exercise's Organization and Relevance

Respondents' perceptions immediately after the training session (Table 4) and after clinical practice (Table 5) were analyzed.

A large proportion of respondents reported that the exercises in the clinical lab were a good way to prepare for the practice. During the exercise, almost half (47%) of them detected that they should have been better prepared. A majority (85%) agreed or strongly agreed that they would be able to master bed bath with a real patient in practice after the training session. Moreover, the respondents' answers from Form 2 were analyzed (Table 5) in relation to the respondents' perceptions after clinical practice.

Experiencing clinical practice strengthened the decision to become a nurse for 95.4% of respondents. When it comes to the question of whether the procedure conducted at the university resembles how it is conducted in clinical practice, 70.6% were undecided or disagreed. Respondents also report that they were confident of mastering a bed bath with a real patient (92% agree/strongly agree).

### 4.2. Comparing the Perceptions of the Two Training Sessions

Based on Questionnaire 1, the groups' beliefs about the teaching program were compared as shown in Table 6.

Both control group and project group seemed to perceive that training sessions in a clinical laboratory were a good way to prepare for practice. However, the project group was significantly less satisfied with the organization of the session than the control group (53.8% versus 92.2% agreed or strongly agree). Respondents from the project group discovered to a significantly greater extent during the exercise that they should have prepared better than the control group (*p* = .013), as 52% agreed or strongly agreed, while 42% in the control group had had this experience. One can also note that 33.9% of respondents in the control group disagreed or strongly disagreed with the statement about preparedness, compared to 17.1% in the project group. A comparison of the groups after clinical practice was done as shown in Table 7.

In total, 158 students carried through clinical practice. Of these, 96 responded (61%), out of which 45% came from the control group and 55% from the project group.

Highest score for both groups was related to the statement that getting out in clinical practice had strengthened their decision to become a nurse. The lowest score was linked to that the procedure in clinical practice resembled the one they had trained for in the laboratory. Still, the majority of both groups were confident that they would master the bed bath with real patients in clinical practice with 97.3% (control group) and 87.2% (project group), respectively, agreeing or strongly agreeing with the statement.

The control group perceived to a significantly larger degree (.002) that the training session for the bed bath provided a good basis for practice.

### 4.3. Comparison of Students with and without Previous Clinical Experience

It was required to see whether the respondents' previous experience with healthcare affected their perceptions of the training session (Table 8). Initially, no significant differences were found between the project group and the control group in regard to the distribution of previous clinical experience.

Respondents with previous clinical experience reported to a significantly larger extent that they were confident to master the bed bath with a real patient in clinical practice, as 95% agreed/strongly agreed with the statement. Among those without any previous clinical practice, 74% reported the same. Out of those who did not have any clinical experience, 78% wanted to seek more information about the procedure, while 60% of those who had clinical experience agreed/strongly agreed with this statement. This difference was significant (.004).

After clinical practice, students with work experience from healthcare were significantly more satisfied with their preparation for practice than those without any experience as 93% and 87%, respectively, agreed/strongly agreed. Other differences emerged (Table 9).

There were also significant differences in the respondents' perceptions of the exercise as a good basis for practice. Sixty-seven percent of the group with work experience agreed/strongly agreed with this statement, compared with 47% of respondents in the group with no previous practice.

### 4.4. Comparing Perceptions Related to Age

There were no significant differences in the project group and control group with respect to range or mean of age. The respondents were divided into three age groups: 19-20 years (Group 1), 21–23 years (Group 2), and >23 years (Group 3). A frequency analysis showed that the oldest group (Group 3) had the fewest number of respondents with previous experience from healthcare. Data from the two surveys were tested by Kruskal–Wallis, and several significant differences between the groups were found. The results indicate that the oldest age group perceived that the training session made them want to seek more knowledge about the procedure to a greater extent (.048) and that they should have spent more time training in the laboratory (.009). After clinical practice, the oldest age group was most confident as far as mastering the bed bath with real patients in clinical practice (.048).

### 4.5. Comparing Perceptions Related to the Amount of Training in the Laboratory

Students were divided into three equal groups associated with the reported time spent on training before practice: 1–4 hours, 5–9 hours, and 10–50 hours. No significant differences between the project group and control group were found in relation to training time, nor were there differences between age groups or in whether the respondents had previous clinical experience regarding time spent on training. The Kruskal–Wallis test showed no significant differences between the training time groups regarding what they reported in either questionnaire 1 or 2.

## 5. Discussion

The findings summarized the students' experiences of comfort and discomfort in the training session, their perceptions of effort and mastery, and their perception of the practical session's usefulness and relevance to clinical practice.

### 5.1. Comfort and Discomfort in the Training Session

The project group were significantly less satisfied with the organization of the training session than the control group. They also did not find the training session as useful as a preparation before clinical practice as did the control group. The organization of the learning activities for the control group's training session was set up according to the principles of traditional model learning [14], as the supervisors actively demonstrated and intervened during the training. As for the project group, the supervisors tried to promote a learning activity that encouraged the students to actively seek solutions to the challenges they faced by being available but withdrawn.

This may have increased the level of frustration in a learning process [15]. Evaluation of learning activities often shows that students are most satisfied with teaching methods in which they adopt a more passive role. As opposed to several studies arguing that student-centered- and active methods provide more meaningful, significant in-depth learning, and long-term learning outcomes [16–18] and that the students' involvement and responsibility for learning are fundamental to good learning outcomes [19]. According to Vygotsky [20], this puts the learner in a place of being in a “discomfort zone.” If this discomfort is manageable, the learning outcome is more integrated and deeper than the case with learning activities that allow the learner to stay in their comfort zone and experience what is called surface learning. This emphasis on the students' own activity and learning outcomes also clearly demands more student preparation before the lesson.

### 5.2. Perceptions of Effort and Mastery

When asked shortly after the training session at the university, an overwhelming majority of the students thought they would be able to master the bed bath in clinical practice.

Students with previous practice were clearly most confident regarding this issue, and all students strengthened this conception after clinical practice. Although some of the differences may be explained by the fact that the response rates to Questionnaires 1 and 2 were 96%–60%, respectively, this is not an unexpected result. More interesting is that there was no difference between the control and project groups' responses to this statement after clinical practice. In other words, clinical practice contributed to evening out the students' confidence regarding mastering a bed bath. These results do not match the results from Struksnes and Engelien [9], who found that the difference between students with and without previous clinical experience lasted throughout clinical practice. However, in the study in question, all students had the same, traditional organization of the training session.

Variables such as students' age, maturity, or educational background may affect the students' sense of achievement and actual performance in relation to the procedure in question.

Concerning age, the students in our project were divided into three age groups. Surprisingly there were more students without clinical practice in the oldest age group, whereas the three age groups had corresponding results before practice. Even so, after the clinical practice the oldest group was more confident in mastering the bed bath in practice than the younger ones. Previous practice from healthcare is considered to be an advantage in nursing education. Our findings indicate that age may compensate for lack of experience. In general, age brings about a cognitive and motoric development that could come into use when practicing nursing skills. Students' age can also have an effect on how and what they learned and seen in relation to the teaching methods that have been used [16, 21]. Andragogic learning is clearly an issue that needs further research, in order to find out more about the connections between age, learning strategies, teaching methods, and learning outcomes.

Altogether, the students expressed immediately after the training session that the practical exercise was useful. Still there were differences between students with or without previous experience from health services, as far as their conception of mastering the bed bath in “real life.” Choice of learning activities aiming at diminishing the difference in confidence between these groups ahead of clinical practice should be explored further. Two alternatives could be interesting: to organize different learning activities for groups with or without previous experience, or to organize the training session as cooperative or social learning, with experienced students learning together with the novices [3].

The extent of rehearsal before practice seemed to have no significance to the students' conception of being confident in mastering the bed bath. Those who before practice were confident in mastering the bed bath correlated with those who believed that practical sessions were good preparation before clinical practice (*r* = .264) and those who thought that the bed bath session at the university provided a good basis for practice (*r* = .279). This indicates that practical sessions at the university before practice strengthen the students' confidence in their mastering of the procedure, although the project group and the control group clearly had different opinions regarding the organization of the practical session at the university.

It is important that the practical sessions at the university encourage the students' sense of empowerment. Research on “meaningful learning” illuminates the significance of involvement, activity, and a sense of achieving the expected learning outcomes as a vital part of the learning process, regardless of age [16, 22]. Nearly half the students discovered during the bed bath session that they should have been better prepared. The project group discovered this to a greater degree than the control group. If students are well prepared, the training session in clinical labs may be used more efficiently. There are differences in students' learning strategies and the amount of time they have to spend to learn. For some, it might be best to prepare ahead of the exercise, as they can prepare at their own pace and manner.

Omitting the film on the web ahead of the training session will also free more time for the students' practical activities during the session. Repetition is a well-known learning principle for manual skills, and according to theories of “learning by doing,” this will increase the level of learning outcomes [23, 24].

Regarding preparations before the internship, the project group discovered to a larger extent than the control group that they should have been prepared for the practical session in the bed bath. However, this experience did not have any impact on how much they prepared on their own before the internship, as the average number of training sessions is very low for the total sample.

There was no difference between the project and control groups in reported training hours before the internship, neither did previous clinical experience, age, or rehearsal time before practice have any significant influence on rehearsal time. Students with previous clinical experience were significantly more satisfied with their own preparation than those without (92.5 and 86.5%, resp.).

There were no differences on how satisfied students were with their own preparation compared to rehearsal time. However, there was broad agreement among the respondents that it is the student's responsibility to practice the procedures before internship. This is in-line with the university's emphasis on students' responsibility to be prepared for the internship.

In summary, there should be more research on the connection between rehearsal time in clinical labs and the student's skills performances in internship.

### 5.3. Perception of Usefulness and Relevance of the Training Session

A large majority of the students thought that CLP was a good way to prepare for the internship, and they also experienced the fact that performing procedures in the internship was easier than expected in advance. This indicates that students were better prepared than they thought and that the training session had fulfilled its intention.

Regarding relevance to clinical practice, the project group agreed with the statement that exercises in the bed bath provided a good basis for practice, although they to a large extent conceived the training session as unsatisfactorily organized.

It is reasonable to assume that the project group with less instruction was more uncertain about the procedure and therefore had to use the time at the training session to familiarize themselves with the procedure. Since there was no difference between the project and control group regarding reported rehearsal time before clinical practice, this may imply that the project group felt more unprepared to perform the procedure in the internship.

One would think that those with previous clinical experience could lean on that experience in the training session and accordingly feel more prepared than they report. This could be explained by the very detailed descriptions of the steps in the procedure, which may seem unfamiliar or irrelevant for someone who has conducted the procedure based on copying experienced employees in the clinical field. Still, with respect to the statements after clinical practice “to conduct the procedure in clinical practice was similar to the one in the training session in the laboratory,” which received a mean score of 3 points (“uncertain”) for the total sample, one could question both the validity and relevance of the content and organization of the training session. Nonetheless, those with previous clinical experience had the highest score on the statement, whether the exercise in the bed bath provided a good basis for practice.

Respondents from control and project group had corresponding opinions about the statement that the training session gave a need for further knowledge, but students without any previous clinical experience tended to score higher on this issue. It is essential that theory and practice are connected during nursing education. Students need a theoretical basis for the clinical procedures, in addition to being able to work in an evidence-based manner. As a nurse, you should be able to verify your actions, and there are increasing demands that practice should be based on knowledge [25, 26].

Those without any previous clinical experience may require a more inductive learning process, or “learning-by-doing” [23, 27], while experienced students reach the level of integrating theory and practice in their clinical performances at an earlier stage. It is debatable whether it is an inductive or deductive way of learning that provides the best learning outcomes, but student activity in the learning process seems to be recommendable, both in the learning of manual skills and cognitive development [28].

Students have different experiences and qualifications to learn, and some students may benefit from studying the procedures themselves in advance, whereas others may learn best by trying procedures first or together with fellow students. Learning activities in higher education, especially those linked to professions where practical skills are implemented in the qualifications for the student to become a competent professional, should enhance social learning strategies and metacognition [29, 30].

There was a positive correlation between those who believed that the practical session for the bed bath initiated a need for knowledge and the fact that being in the practice field had strengthened the decision to become a nurse (*r* = .365). Those without any clinical experience were more likely to seek knowledge. However, both groups equally stated that being in the period of internship strengthened the decision to become a nurse.

## 6. Conclusions

The majority of the nursing students reported that the training sessions were useful and were confident to master bed bath in clinical practice after the skills training session. Almost half the students detected on the way that they should have been better prepared, and most of them did not perceive that the procedure conducted at the university resembles how it is conducted in clinical practice.

The project group was significantly less satisfied with the organization of the session than the control group, while the control group perceived to a significantly larger degree that the training session for the bed bath provided a good basis for practice.

Before the internship, students with previous clinical experience were most confident to master the bed bath with a real patient in clinical practice, but after the internship the difference between the groups evened out.

Those who did not have any clinical experience to a larger extent considered the exercise as a good basis for practice.

Students with work experience from healthcare were most satisfied with their preparation for practice.

The oldest age group were more aware that they should have spent more time training in the laboratory, and they were most confident as far as mastering bed bath. The results from this study could indicate that the universities' facilitation of nursing students' clinical preparations may have an impact on the students' feeling of mastery and confidence before their internship. Organization of the training session or previous experience does not seem to have any significant impact, but age and experiences in the internship seem to influence the learning outcome. Thus, these findings should be considered in the continuous work in the educational institutions, to improve the transition to “real life” as smoothly and meaningfully as possible to nursing students.

## Figures and Tables

**Table 1:** Questionnaires.

After training session	After clinical practice
Before the training session I was well prepared by reading the Procedures in Clinical Practice in Nursing© (PCPN)	We should have more time to practice in the laboratory
The training session was organized in a good way	I am content with my preparedness to conduct the procedure before clinical practice
I discovered during the session that I should have prepared myself better	The training session contributed to a good basis for conducting the same in clinical practice
Students in my group used the training time effectively	It was easier than expected to perform the procedure in clinical practice
I'm confident that I will master the bed bath with a real patient in clinical practice	To conduct the procedure in clinical practice was similar to the one in the training session in the laboratory
The training session made me want to seek more knowledge about the procedure	I am confident that I will master the procedure with a real patient in clinical practice
It's my responsibility as a student to practice the procedures until practiced	The internship has strengthened my decision to become a nurse
Training sessions in the laboratory is a good way to prepare oneself for clinical practice	

**Table 2:** Elements of the implementation of the two groups.

	*Control group (CG)* (Class 1, *N* = 79)	*Project group (PG)* (Class 2, *N* = 81)
Written and oral information about the practical implementation in advance	✓	✓
Encouraging preparation before the training session	✓	✓
Video viewing before the training session	✓	O
Information about organization	✓	✓
Supervisor resources	1 pr. group (8–12)	1 pr. two groups (16–24)
Supervisor's role	Active participation with specific supervision	Available only for practical arrangements and equipment supply
Services on hardcopy of (PCPN)	O	✓

**Table 3:** Respondents.

*Questionnaire 1, after training session* *N* = 160		*Questionnaire 2, after clinical practice* *N* = 158	
*n* (%)	154 (96)	*n* (%)	96 (61)
Former practice	82	Former practice	55
Not earlier practice	69	Not earlier practice	38

**Table 4:** Perceptions of all respondents immediately after the training session (Questionnaire 1).

*N* = 160	*n* (%)	Mean	SD
Before the training session I was well prepared by reading the PCPN	154 (96)	3.36	1.181
The training session was organized in a good way	154 (96)	3.72	.889
I discovered during the session that I should have prepared myself better	153 (96)	3.31	1.096
Students in my group used the training time effectively	153 (96)	4.24	.744
I'm confident that I will master the bed bath with a real patient in clinical practice	154 (96)	4.05	.851
The training session made me want to seek more knowledge about the procedure	154 (96)	3.81	.831
It's my responsibility as a student to practice the procedures until practiced	154 (96)	4.65	.578
Training sessions in the laboratory is a good way to prepare oneself for clinical practice	154 (96)	4.82	.414

Scale: 1 = strongly disagree, 2 = disagree, 3 = unsure, 4 = agree, and 5 = strongly agree.

**Table 5:** Perceptions of all respondents, after clinical practice (Questionnaire 2).

*N* = 158	*n* (%)	Mean	SD
We should have more time to practice in the laboratory	95 (60)	3.26	1031
I am content with my preparedness to conduct the procedure before clinical practice	96 (61)	3.99	.584
The training session contributed to a good basis for conducting the same in clinical practice	94 (60)	3.46	1103
It was easier than expected to perform the procedure in clinical practice	87 (55)	3.88	.817
To conduct the procedure in clinical practice was similar to the one in the training session in the laboratory	85 (54)	2.98	.922
I am confident that I will master the procedure with a real patient in clinical practice	88 (56)	4.23	.797
The internship has strengthened my decision to become a nurse	88 (56)	4.55	.589

Scale: 1 = strongly disagree, 2 = disagree, 3 = unsure, 4 = agree, and 5 = strongly agree.

**Table 6:** Comparison of control and project group, after training session.

	Total *N* = 160			CG *n* = 79			PG *n* = 81			Mann–Whitney *U* test, (2-tailed)	
	*n* (%)	Mean	SD	*n* (%)	Mean	SD	*n* (%)	Mean	SD	*Z*	*p*
Before the training session I was well prepared by reading the PCPN	154 (96)	3.36	1,181	76 (96)	3.49	1,172	78 (96)	3.23	1,183	1,377	.169
The training session was organized in a good way	154 (96)	3.72	.889	76 (96)	4.13	.525	78 (96)	3.32	.987	5,521	.000
I discovered during the session that I should have prepared myself better	153 (96)	3.31	1,096	76 (96)	3.07	1,112	77 (95)	3.55	1,033	2472	.013
Students in my group used the training time effectively	153 (96)	4.24	.744	75 (95)	4.29	.693	78 (96)	4.19	.790	−.685	.493
I'm confident that I will master the bed bath with a real patient in clinical practice	154 (96)	4.05	.851	76 (96)	4.17	.700	78 (96)	3.92	.964	1,466	.143
The training session made me want to seek more knowledge about the procedure	154 (96)	3.81	.831	76 (96)	3.74	.789	78 (96)	3.88	.868	1,380	.168
It's my responsibility as a student to practice the procedures until practiced	154 (96)	4.65	.578	76 (96)	4.67	.551	78 (96)	4.63	.605	−.400	.689
Training sessions in the laboratory is a good way to prepare oneself for clinical practice	154 (96)	4.82	.414	76 (96)	4.87	.377	78 (96)	4.78	.446	1,433	.152

Scale: 1 = strongly disagree, 2 = disagree, 3 = unsure, 4 = agree, and 5 = strongly agree.

**Table 7:** Comparison of control and project group, after clinical practice (Questionnaire 2).

	Total *N* = 158			CG *n* = 78			PG *n* = 80			Mann–Whitney *U* test, 2-tailed	
	*n* (%)	Mean	SD	*n* (%)	Mean	SD	*n* (%)	Mean	SD	*Z*	*p*
We should have more time to practice in the laboratory	95 (60)	3.26	1,044	42 (54)	3.10	1135	53 (66)	3.42	.949	1.378	.168
I am content with my preparedness to conduct the procedure before clinical practice	96 (61)	3.98	.580	43 (55)	4.07	.402	53 (66)	3.91	.687	1.085	.278
The training session contributed to a good basis for conducting the same in clinical practice	94 (60)	3.47	1,104	42 (54)	3.88	.832	52 (65)	3.13	1.189	3.104	.002
It was easier than expected to perform the procedure in clinical practice	87 (55)	3.87	.804	39 (50)	3.79	.951	48 (60)	3.94	.665	−.555	.579
To conduct the procedure in clinical practice was similar to the one in the training session in the laboratory	85 (54)	2.94	.930	37 (47)	3.00	.943	48 (60)	2.90	.928	−.684	.494
I am confident that I will master the procedure with a real patient in clinical practice	88 (56)	4.22	.780	39 (50)	4.41	.559	49 (61)	4.06	.899	1849	.064
The internship has strengthened my decision to become a nurse	88 (56)	4.56	.584	39 (50)	4.56	.598	49 (61)	4.55	.580	−.171	.864

Scale: 1 = strongly disagree, 2 = disagree, 3 = unsure, 4 = agree, and 5 = strongly agree.

**Table 8:** Comparison of students with and without previous practice in healthcare, after training session.

*N* = 160	Total *n* = 151			Not practice *n* = 69			Practice *n* = 82			Mann–Whitney *U* test 2-tailed	
	*n* (%)	Mean	SD	*n* (%)	Mean	SD	*n* (%)	Mean	SD	*Z*	*p*
Before the training session I was well prepared by reading the PCPN	151 (94)	3.34	1,183	69 (100)	3.51	1,171	82 (100)	3.20	1,180	1,705	.088
The training session was organized in a good way	151 (94)	3.71	.884	69 (100)	3.74	.885	82 (100)	3.68	.887	−.335	.737
I discovered during the session that I should have prepared myself better	150 (94)	3.29	1,096	69 (100)	3.45	1,119	81 (99)	3.16	1,066	1,603	.109
Students in my group used the training time effectively	150 (94)	4.23	.743	68 (99)	4.22	.666	82 (100)	4.23	.806	−.520	.603
I'm confident that I will master the bed bath with a real patient in clinical practice	151 (94)	4.04	.848	69 (100)	3.71	1,001	82 (100)	4.32	.564	4,195	.000
The training session made me want to seek more knowledge about the procedure	151 (94)	3.81	.836	69 (100)	4.04	.695	82 (100)	3.62	.898	2,849	.004
It's my responsibility as a student to practice the procedures until practiced	151 (94)	4.64	.581	69 (100)	4.57	.630	82 (100)	4.71	.533	1,551	.121
Training sessions in the laboratory is a good way to prepare oneself for clinical practice	151 (94)	4.82	.418	69 (100)	4.83	.382	82 (100)	4.82	.448	−.183	.855

Scale: 1 = strongly disagree, 2 = disagree, 3 = unsure, 4 = agree, and 5 = strongly agree.

**Table 9:** Comparison of students with and without previous practice in healthcare, after clinical practice.

	Total *N* = 96			Not practice *n* = 38			Practice *n* = 55			Mann–Whitney *U* test 2-tailed	
	*n* (%)	Mean	SD	*n* (%)	Mean	SD	*n* (%)	Mean	SD	*Z*	*p*
We should have more time to practice in the laboratory	92 (96)	3.26	1,039	38 (100)	3.35	1033	54 (98)	3.19	1049	−.857	.391
I am content with my preparedness to conduct the procedure before clinical practice	93 (97)	3.99	.571	38 (100)	3.84	.442	55 (100)	4.09	.628	2614	.009
The training session contributed to a good basis for conducting the same in clinical practice	91 (95)	3.47	1,114	37 (98)	3.17	1159	54 (98)	3.67	1,043	2,109	.035
It was easier than expected to perform the procedure in clinical practice	85 (89)	3.88	.822	35 (92)	3.68	.945	50 (91)	4.02	.699	1,487	.137
To conduct the procedure in clinical practice was similar to the one in the training session in the laboratory	83 (87)	2.96	.920	33 (87)	2.75	.880	50 (91)	3.10	.928	1,745	.081
I am confident that I will master the procedure with a real patient in clinical practice	86 (90)	4.24	.790	35 (92)	4.29	.524	51 (93)	4.20	.935	−.273	.785
The internship has strengthened my decision to become a nurse	86 (90)	4.54	.591	35 (92)	4.41	.609	51 (93)	4.63	.566	1,789	.074
